# Impact of code stroke on door-to-andexanet administration time for factor Xa inhibitor-associated intracranial hemorrhage: a single-center retrospective study

**DOI:** 10.3389/fneur.2026.1765311

**Published:** 2026-03-25

**Authors:** Koji Tanaka, Eri Imazu, Takuma Ishihara, Junpei Koge, Tetsuya Hashimoto, Kazuyasu Matsumura, Eiji Fujiwara, Kenichiro Suyama, Jun Muto, Motoharu Hayakawa, Ayuko Yasuda, Ichiro Nakahara, Hiroshi Koyama, Jun-ichi Kira, Yuichi Hirose, Shoji Matsumoto

**Affiliations:** 1 Department of Comprehensive Strokology, Fujita Health University, Toyoake, Japan; 2 Fujita Health University School of Medicine, Toyoake, Japan; 3 Innovative and Clinical Research Promotion Center, Gifu University Hospital, Gifu, Japan; 4 Department of Neurosurgery, Fujita Health University, Toyoake, Japan; 5 Department of Neurosurgery, Fujita Health University Okazaki Medical Center, Okazaki, Japan; 6 Department of Patient Safety and Quality Management, National Hospital Organization Nagoya Medical Center, Nagoya, Japan; 7 Department of Neurosurgery, Fujita Health University Bantane Hospital, Nagoya, Japan; 8 Graduate School of Industrial Technology, Advanced Institute of Industrial Technology, Tokyo, Japan; 9 Translational Neuroscience Research Center, Graduate School of Medicine, International University of Health and Welfare, Okawa, Japan; 10 Department of Neurology, Brain and Nerve Center, Fukuoka Central Hospital, Fukuoka, Japan

**Keywords:** andexanet alfa, code stroke, factor Xa inhibitor, intracranial hemorrhage, treatment time

## Abstract

**Background:**

Administration of andexanet alfa has shown to achieve hemostatic efficacy in factor Xa inhibitor (FXai)-associated intracranial hemorrhage (ICrH). Code stroke (CS), implemented through the visual task management application Task Calc. Stroke (TCS) facilitates timely reperfusion therapy for acute ischemic stroke. However, the association between TCS-based CS and in-hospital treatment time of andexanet for FXai-associated ICrH remains unknown.

**Methods:**

In this single-center retrospective study, patients with FXai-associated ICrH who received andexanet were enrolled from May 2022 to May 2025. TCS was activated via prehospital notification when patients presented with at least one of the clinical symptoms including face dropping, arm weakness, or speech difficulty with a time from onset or last known well of <24 h. Multivariable linear regression was performed to investigate the association between TCS-based CS and door-to-andexanet administration time.

**Results:**

Forty-two patients (22 men, median age 80 years) were included. The primary location of hemorrhage was intracerebral (*n* = 26), epidural/subdural (*n* = 8), or subarachnoid (*n* = 8). Among them, 17 (41.5%) were treated with TCS-based CS. The door-to-andexanet administration time was shorter in patients treated with TCS-based CS compared to those without (90 min vs. 132 min, *p* < 0.01). Multivariable analysis showed that TCS-based CS was associated with door-to-andexanet administration time (Exp [*β*] 0.58, 95% confidence interval 0.43–0.77) after adjustment with arrival during regular hours and baseline hematoma volume.

**Conclusion:**

TCS-based CS was associated with a shorter door-to-andexanet administration time for FXai-associated ICrH. The outcome benefit from improved treatment times warrants further investigation.

## Introduction

Intracranial hemorrhage (ICrH) including intracerebral hemorrhage (ICH) is a life-threatening condition associated with high mortality and severe long-term disability (1). Hematoma expansion (HE) is a major contributor to poor outcomes in patients with ICrH (2, 3), and those taking anticoagulants are at high risk of HE (4, 5). Administration of andexanet alfa, a reversal agent for factor Xa inhibitor (FXai), has demonstrated hemostatic efficacy in patients with FXai-associated ICrH (6, 7). Given that most HE occurs shortly after the onset (8), early hemostatic treatment would be crucial to mitigating further bleeding and improving outcomes.

The Task Calculation Stroke (Task Calc. Stroke: TCS) is a visual task management application to shorten in-hospital workflow times for acute reperfusion therapy (i.e., intravenous thrombolysis and mechanical thrombectomy) in patients with acute ischemic stroke by facilitating parallel task processing among medical staff (9). Previous studies have shown that TCS-based code stroke (TCS-based CS) could significantly shorten the key in-hospital time metrics, including door-to-imaging, door-to-needle, and door-to-puncture times in the management of acute ischemic stroke (9, 10).

Because TCS is activated via prehospital notification, it can be also activated for patients with FXai-associated ICrH. Although TCS is primarily designed to streamline the management of acute ischemic stroke, the activation of TCS may also contribute to a shorter in-hospital workflow for andexanet administration in patients with FXai-associated ICrH. Therefore, in this study, we aimed to investigate the impact of TCS-based CS on the in-hospital treatment time of andexanet in patients with FXai-associated ICrH.

## Materials and methods

### Study design

This is a single-center retrospective cohort study consisting of consecutive patients who received andexanet (Ondexxya^Ⓡ^) for FXai-associated ICrH from 25th May 2022 (andexanet has become available for use in Japan) to 31st May 2025. Patients with FXai-associated ICrH who developed hemorrhage during hospitalization or who presented as walk-in arrivals were excluded. TCS was implemented in our hospital from June 2018. The details of TCS have been described elsewhere (9, 10). Briefly, TCS is a mobile application for stroke care management that can be used on commercially available smartphones or tablets via the internet. TCS has 4 important features: (1) the notification feature alerts staff about patient arrival, (2) a synchronous timer feature enables all team members to estimate the start time of their tasks for the target patient, (3) the visual task management feature shares the tasks required among team members and tracks parallel completion, and (4) the history analysis feature monitors team performance to identify areas of improvement by real-time feedback. We did not do real-time feedback for the time of andexanet administration throughout the study period. Using the performance history analysis function of TCS (9), we investigated the proportion of patients who received andexanet for FXai-associated ICrH among patients for whom TCS was activated during the study period. The decision to activate TCS was made by the attending emergency physician in consultation with a neurologist or neurosurgeon, based on prehospital information provided by emergency medical services. Eligibility for TCS activation required the presence of at least one of the following symptoms: facial drop, arm weakness, or speech disturbance, with a time from onset or last known well of less than 24 h. The decision to administer andexanet was made by the attending neurologist or neurosurgeon. There was no dedicated protocol or checklist for andexanet administration. Once andexanet was prescribed, the drug was dispensed from the storage area, delivered to the emergency room, and prepared for administration by nurses or pharmacists. Patients received either a high-dose or a low-dose bolus over 15 to 30 min, followed by a continuous infusion over 2 h. The use of a high-dose or low-dose bolus was in accordance with the labeling approved by the Pharmaceuticals and Medical Devices Agency and was based on the type, dose, and timing of the most recent FXai that was received. A schematic workflow of andexanet administration under TCS-based CS has been added as Supplementary Figure 1. We used the STROBE reporting guideline (11) to draft this manuscript, and the STROBE reporting checklist (12) when editing.

### Study participants

The following clinical information was collected from the emergency medical charts: sex, age, prehospital Glasgow Coma Scale (GCS), history of preceding trauma, indication for FXai (atrial fibrillation or deep-vein thrombosis), time of arrival (regular hours [8 a.m. to 5 p.m. on weekdays] or off-hours [5 p.m. to 8 a.m. on weekdays and weekends]), systolic and diastolic blood pressure on admission, and administered dose of andexanet. Time-related metrics, including onset-to-door time, door-to-imaging time, and door-to-andexanet administration time were calculated. The door-to-imaging time reflects CT acquisition and does not include interpretation time. Door-to-complete blood count time, which was one of the predefined TCS metrics, was available only for patients treated under TCS. The National Institutes of Health Stroke Scale (NIHSS) was assessed in patients with ICH.

### Imaging analysis

The primary location of hemorrhage was categorized by noncontrast CT (NCCT) on admission as intracerebral (ICH), subarachnoid, or subdural/epidural. Hematoma volumes on baseline and follow-up NCCT were measured on 5-mm axial slices using the ABC/2 method, as previously described (13–15). Radiographic hemostatic efficacy was defined as excellent or good (≤20% or ≤35% increase in hematoma volume from baseline, respectively) (16). Patients who underwent hematoma evacuation before follow-up imaging were excluded from the assessment of radiographic hemostatic efficacy.

### Statistical analysis

All statistical analyses were performed with Stata statistical software version 18 (Stata Corp, College Station, TX). Data were described as median values with interquartile range (IQR) for continuous variables and counts and percentages for categorical variables. Baseline characteristics and time metrics were compared between patients treated under TCS-based CS or not using the chi-square test, Fisher exact test, or Wilcoxon rank sum test as appropriate. To investigate factors influencing the decision to activate or withhold TCS, variables potentially available in the prehospital setting including history of preceding trauma, prehospital GCS, FXai use, and time of arrival were compared between patients treated under TCS-based CS or not, regardless of andexanet administration. An analysis of covariance using a linear regression model was performed to determine the reduction effect of TCS-based CS on door-to-andexanet administration. Although the systematic bias present in TCS-based CS and door-to-andexanet administration was considered to be negligible, the number of covariates was restricted to avoid model overfitting (17). We employed two variables: time of arrival, an established factor for delay in thrombolysis in acute ischemic stroke, as an off-hour effect (18, 19); and hematoma volume, which may affect treatment decision-making regarding adexanet. Given their clinical importance, we performed additional analyses by adding history of preceding trauma and prehospital GCS as additional covariables in the multivariable model. Due to the non-normal distribution of door-to-andexanet administration time and the potential violation of the normality assumption of model residuals, a logarithmic transformation was applied prior to analysis. For presentation of the results, back-transformation using the exponential function was performed. Predicted values obtained from the model with covariates fixed at the median were plotted. A similar modeling was performed for door-to-imaging time. An exploratory analysis was performed to compare the frequency of radiographic hemostatic efficacy between patients treated under TCS-based CS or not. A two-sided *p* value of <0.05 was considered statistically significant.

## Results

Among a total of 76 patients with direct oral anticoagulants-associated ICrH, there were 24 patients with FXai-associated ICrH for whom TCS was activated, accounting for 2.3% of the 1,026 patients with suspected acute stroke who underwent TCS activation. One patient with ICrH was taking dabigatran, and no patients received prothrombin complex concentrate (PCC) as an alternative to andexanet during the study period. Patients’ characteristics in those treated under TCS-based CS or not, regardless of andexanet administration, are shown in Supplementary Table 1. Among 42 patients (22 men, median age of 80 years) included, 17 patients received andexanet under TCS-based CS, while 25 patients received andexanet without TCS-based CS (Supplementary Figure 2). The primary location of hemorrhage was intracerebral (*n* = 26), followed by epidural or subdural (*n* = 8), or subarachnoid (*n* = 8). The median hematoma volume was 15.7 (IQR 3.4–68.1) ml and 19 patients (45%) arrived during the regular hours. Among patients with ICH, the median NIHSS was 12 (IQR 3–22).

Patients’ characteristics in those treated under TCS-based CS or not were shown in Table 1. Patients treated under TCS-based CS were less likely to have a history of preceding trauma (12% vs. 56%, *p* < 0.01) than those without. The proportion of hemorrhage type was different between the groups (*p* = 0.02): patients treated under TCS-based CS were more likely to have ICH (88%) than those without (44%). Time-related metrics of andexanet administration were shown in Table 2. Door-to-imaging and door-to-andexanet administration times were shorter in patients treated under TCS-based CS than those without (median 10 min vs. 20 min, *p* < 0.01; and median 90 min vs. 132 min, *p* < 0.01, respectively).

**Table 1:** Patients’ characteristics.

Variable	TCS-based CS		*p-*value
	Yes (*n* = 17)	No (*n* = 25)	
Sex, male	12 (71)	10 (40)	0.07
Age in years	78 (74–84)	82 (77–91)	0.09
Body mass index	22 (20–27)	21 (19–24)	0.27
Prehospital GCS	13 (8–14)	14 (12–15)	0.07
History of preceding trauma	2 (12)	14 (56)	<0.01
Treatment target disease of FXai			0.88
Atrial fibrillation	12 (71)	19 (76)	
Deep-vein thrombosis	3 (18)	3 (12)	
Other/unclear	2 (12)	3 (12)	
FXai use			0.30
Apixaban	3 (18)	4 (16)	
Rivaroxaban	1 (6)	6 (24)	
Edoxaban	13 (76)	15 (60)	
Arrival time, regular hours	9 (53)	10 (40)	1.00
Systolic blood pressure, mmHg	179 (152–204)	172 (146–179)	0.11
Diastolic blood pressure, mmHg	105 (86–111)	92 (80–107)	0.22
Primary hemorrhage location			0.02
Intracerebral	15 (88)	11 (44)	
Subarachnoid	1 (6)	7 (28)	
Subdural or epidural	1 (6)	7 (28)	
Hematoma volume, mL	40.6 (10.8–98.0)	9.8 (1.0–60.5)	0.12
High dose eligible population	9 (53)	9 (36)	0.35

Data are presented as *n* (%) or median (interquartile range). CS, code stroke; FXai, factor Xa inhibitor; GCS, Glasgow Coma Scale; TCS, Task Calculation Stroke.

**Table 2:** Time-related metrics of andexanet administration.

Variable	TCS-based CS		*p*-value
	Yes (*n* = 17)	No (*n* = 25)	
Onset-to-door time, min^*^	148 (52–442)	102 (50–430)	0.64
Door-to-imaging time, min	10 (5–15)	20 (13–40)	<0.01
Door-to-CBC time, min^†^	14 (8–20)	–	–
Imaging-to-andexanet administration time, min	69 (62–102)	100 (67–151)	0.06
Door-to-andexanet administration time, min	90 (73–109)	132 (99–195)	<0.01
Onset-to-andexanet administration time, min^*^	228 (132–500)	292 (180–576)	0.23

Data are presented as median (interquartile range). CBC, complete blood count; CS, code stroke; TCS, Task Calculation Stroke. ^*^Onset-to-door and onset-to-andexanet administration times were available for 39 patients. ^†^Door-to-CBC time was available only in patients with TCS-based CS.

**Figure 1:**
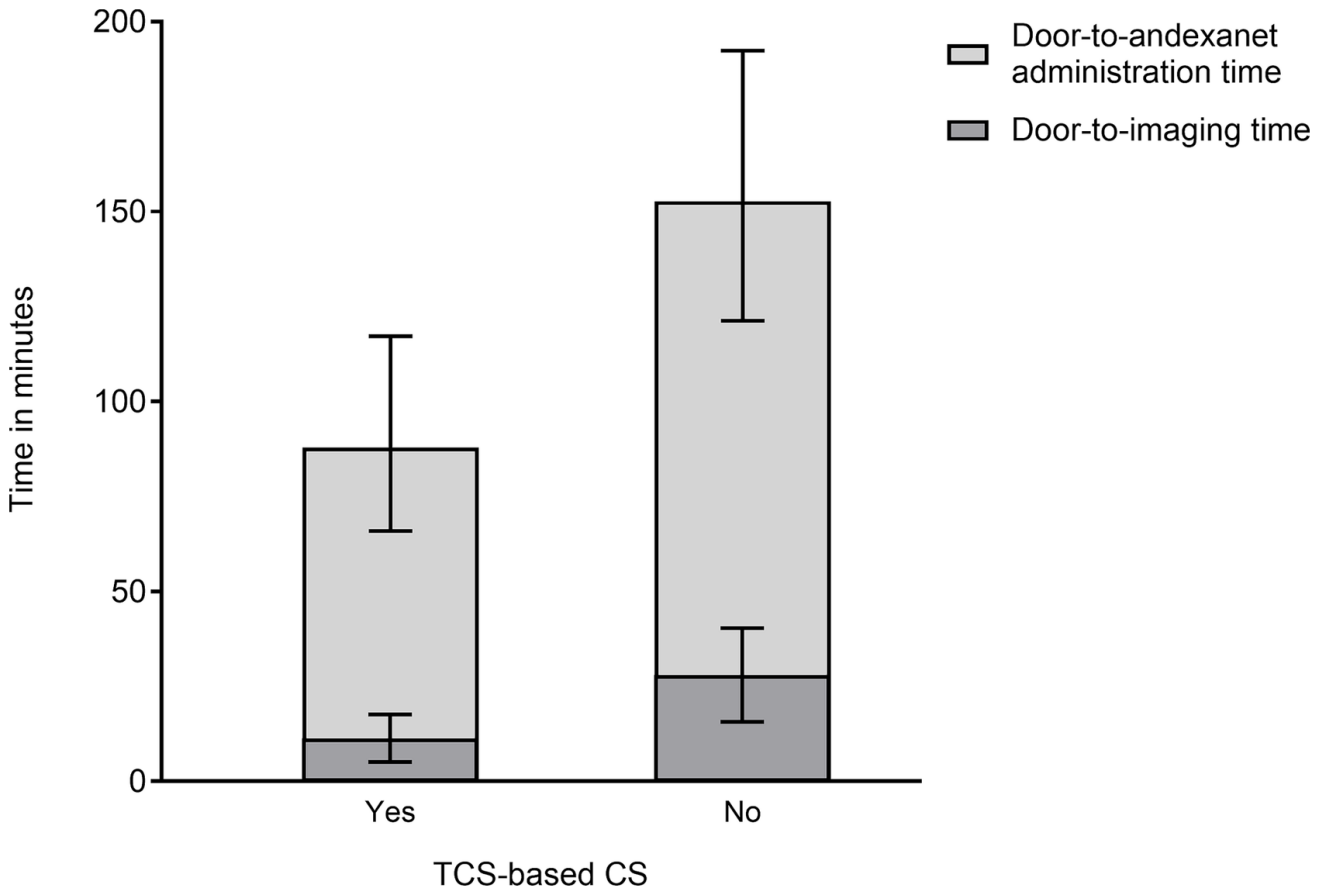
Estimated door-to-imaging and door-to-andexanet administration times in patients with and without TCS-based CS. Predicted values for door-to-andexanet administration time were output with the covariates fixed at the mode and median values, i.e., off-hours, and hematoma volume of 14.4 mL. Predicted values for door-to-imaging time were output with the time of arrival. The points represent the predicted mean, and the bars indicate the 95% confidence interval. CS, code stroke; TCS, task calculation stroke.

The multivariable linear regression analysis showed that TCS-based CS was associated with door-to-andexanet administration time (Exp [*β*] 0.58, 95% confidence interval [CI] 0.43–0.77, p < 0.01) after adjustment with arrival time and hematoma volume (Table 3). TCS-based CS was also associated with door-to-imaging time (Exp [*β*] 0.40, 95% CI 0.22–0.70, p < 0.01). The result was consistent with the primary analysis when including history of preceding trauma and prehospital GCS as additional covariables (Supplementary Table 2). The modeling showed that an estimated mean door-to-imaging time was 10.5 (95% CI 6.0–18.4) minutes in patients treated under TCS-based CS and 26.5 (95% CI 16.9–41.5) minutes in those without; and door-to-andexanet administration time was 87.8 (95% CI 65.9–117.2) minutes in patients treated under TCS-based CS and 152.7 (95% CI 121.3–192.4) minutes in those without (Figure 1).

**Table 3:** The effect of TCS-based CS on door-to-andexanet administration time.

Variable	Exp (*β*)	95% CI	*p*-value
TCS-based CS (yes)	0.58	0.43–0.77	<0.01
Arrival time (regular hours)	0.72	0.54–0.96	0.03
Hematoma volume (per 10-ml increase)	1.02	1.00–1.04	0.06

Exp (*β*) indicates the rate of decrease in door-to-andexanet administration time. CI, confidence interval; CS, code stroke; TCS, Task Calculation Stroke.

Follow-up imaging was available for 35 patients and was performed a median of 17.1 h after andexanet administration. There were no statistically significant differences in excellent hemostatic efficacy (73% vs. 55%, *p* = 0.31) or good hemostatic efficacy (80% vs. 60%, *p* = 0.28) between patients treated under TCS-based CS or not.

## Discussion

In the present study of patients who received andexanet for FXai-associated ICrH, those treated under TCS-based CS were less likely to have a history of preceding trauma compared to those treated without TCS-based CS. The door-to-andexanet administration time was shorter in patients managed under TCS-based CS, and multivariable analysis showed that TCS-based CS was associated with a shorter door-to-andexanet administration time.

TCS was developed to facilitate parallel task completion among stroke center staff by supporting communication and coordination (9, 10). Timely brain imaging enabled by TCS-based CS, evidenced by a shorter door-to-imaging time, might help clinicians in timely clinical decision-making and treatment. Notably, pharmacists were integrated into the stroke team and received real-time notifications via TCS. Previous studies have shown that pharmacist involvement in emergency departments contributes not only to the timely administration of critical therapies, including intravenous thrombolysis and anticoagulant reversal (20–22), but also to reduce medical errors (23). This involvement allows clinicians to focus more on treatment decisions rather than medication retrieval or preparation.

Because TCS was not randomly applied, several clinical factors might have influenced TCS activation or withholding. Patients presenting with more severe neurological deficits or ischemic stroke-like symptoms may have been more likely to trigger pre-hospital notification and TCS activation. In this study, patients treated under TCS-based CS were less likely to have a history of preceding trauma, suggesting that clinicians might have withheld TCS activation when prehospital information indicated recent trauma. This is consistent with current guidelines for the early management of patients with acute ischemic stroke, which consider significant trauma an exclusion criterion for administering recombinant tissue-type plasminogen activator (24, 25). Moreover, patients with trauma often require wound management in addition to the initial neurological evaluation, which might delay the in-hospital workflow. Other unmeasured factors might also have independently affected workflow efficiency. Notably, TCS was not activated for more than half of patients with FXai-associated ICrH who received andexanet, and only 2.3% of TCS activations involved patients with FXai-associated ICrH. These findings suggest that the current FAST (Face, Arm, Speech, Time) algorithm may be insufficient for identifying patients with FXai-associated ICrH who are eligible for hemostatic treatment. Incorporating additional prehospital information such as anticoagulant use, regardless of history of preceding trauma, may improve the screening and triage of these patients (26), although prehospital information may often be insufficient in the emergency medical care setting. Recent scientific data have demonstrated promising results from novel emergency interventions for ICH and a set of specific time-based quality metrics for emergency ICH care, “Code ICH,” has been proposed (27–29). Our findings suggest that TCS can streamline in-hospital workflows not only for acute ischemic stroke and FXai-associated ICrH, but also for other stroke types that require timely interventions.

This study has several limitations. First, this is a single center retrospective study with a small sample size, and the treatment decision regarding TCS activation and andexanet administration was made by attending clinicians, potentially introducing selection bias. Due to its cost and availability, clinicians might withhold treatment in patients who had taken FXai well beyond their expected half-life. Moreover, TCS is an institution-specific system and may not be directly applicable to other CS systems. Second, walk-in or in-hospital ICrH cases, in which delays in diagnosis and treatment may be most pronounced, were excluded. Factors affecting time-related metrics of andexanet administration in these populations remain unclear. Third, because there were few or no patients with ICrH who were taking dabigatran or receiving PCC, we could not investigate the impact of TCS-based CS on other hemostatic treatments. Fourth, the ABC/2 method was not applicable to intraventricular hemorrhage, and therefore, we excluded intraventricular hematoma from the hematoma volume calculation. Finally, the difference in the frequency of radiographic hemostatic efficacy was not significant between patients treated under TCS-based CS or not, and this study did not assess functional outcomes. Because the processes and treatment decisions were multifactorial, this study could not clarify which steps (e.g., uncertainty about the indication, varying risk–benefit assessments, or delays in obtaining approval or consent) were directly influenced by TCS. Further multicenter studies are warranted to validate the generalizability of TCS-based CS, investigate the impact of additional clinical factors on the association between TCS-based CS and time-related metrics, and determine whether shortening the door-to-andexanet administration time can help prevent HE and improve outcomes in patients with FXai-associated ICrH.

## Conclusion

TCS-based CS was associated with a shorter door-to-andexanet administration time in ambulance-transported patients with FXai-associated ICrH. The outcome benefit from improved treatment times warrants further investigation.

## Data Availability

The raw data supporting the conclusions of this article will be made available by the authors, without undue reservation.

## Supplementary material

Supplementary Figure 1 Workflow of the andexanet administration under TCS-based CS CS, code stroke; EMS, emergency medical service; FXai, Factor Xa inhibitor; ICrH, intracranial hemorrhage; TCS, Task Calculation Stroke.

Supplementary Figure 2 Study flowchart. DOAC, direct oral anticoagulant; FXai, Factor Xa inhibitor; ICrH, intracranial hemorrhage; TCS, Task Calculation Stroke.
