# Supplementary figures and images for: Impact of code stroke on door-to-andexanet administration time for factor Xa inhibitor-associated intracranial hemorrhage: a single-center retrospective study
Source: Front Neurol. 2026 Mar 25;17:1765311. doi: 10.3389/fneur.2026.1765311 (PMC13056841; doi:10.3389/fneur.2026.1765311)

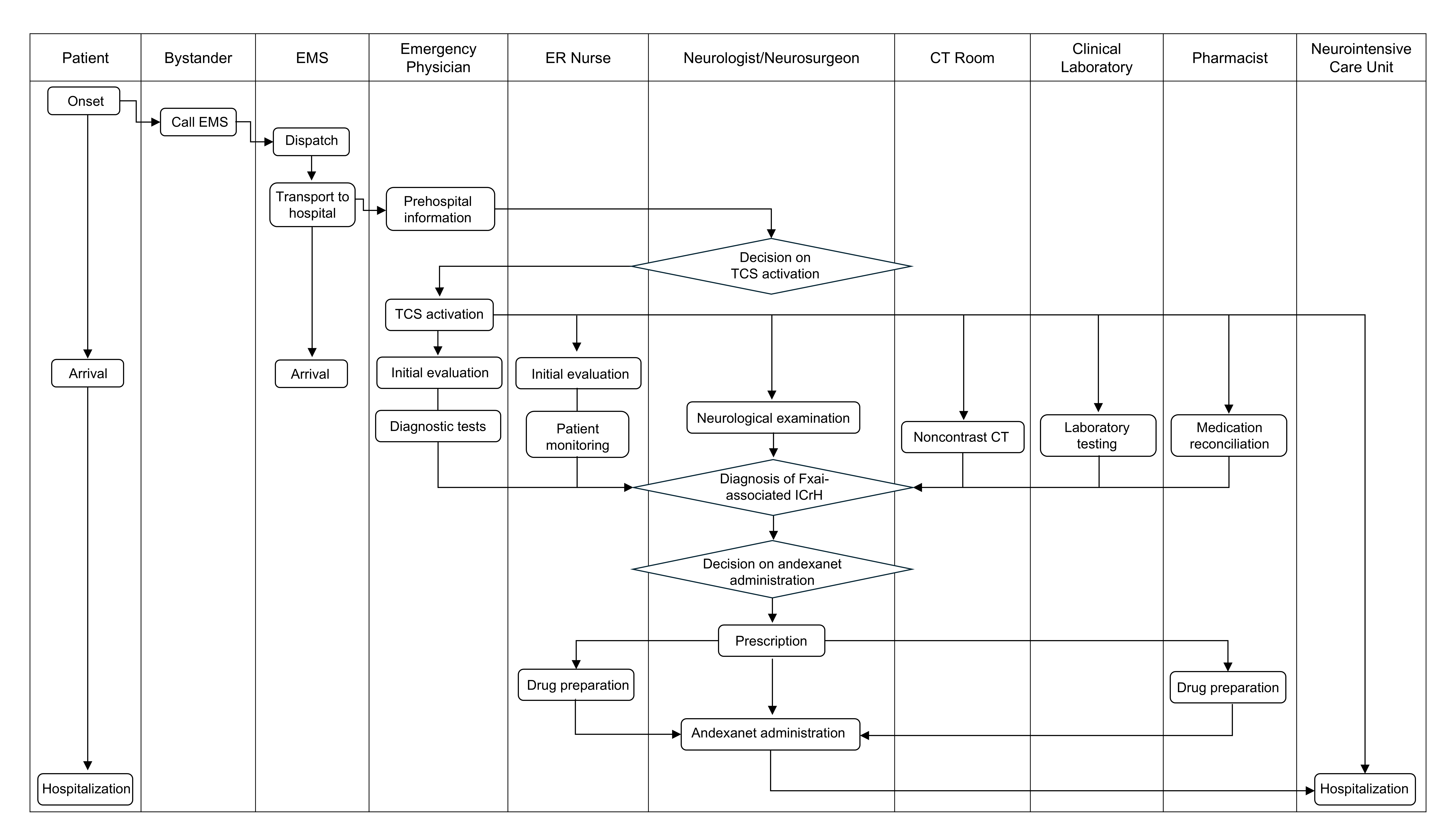

Supplement: Supplementary Figure 1 — Workflow of the andexanet administration under TCS-based CS CS, code stroke; EMS, emergency medical service; FXai, Factor Xa inhibitor; ICrH, intracranial hemorrhage; TCS, Task Calculation Stroke. [file Image_1.tif]

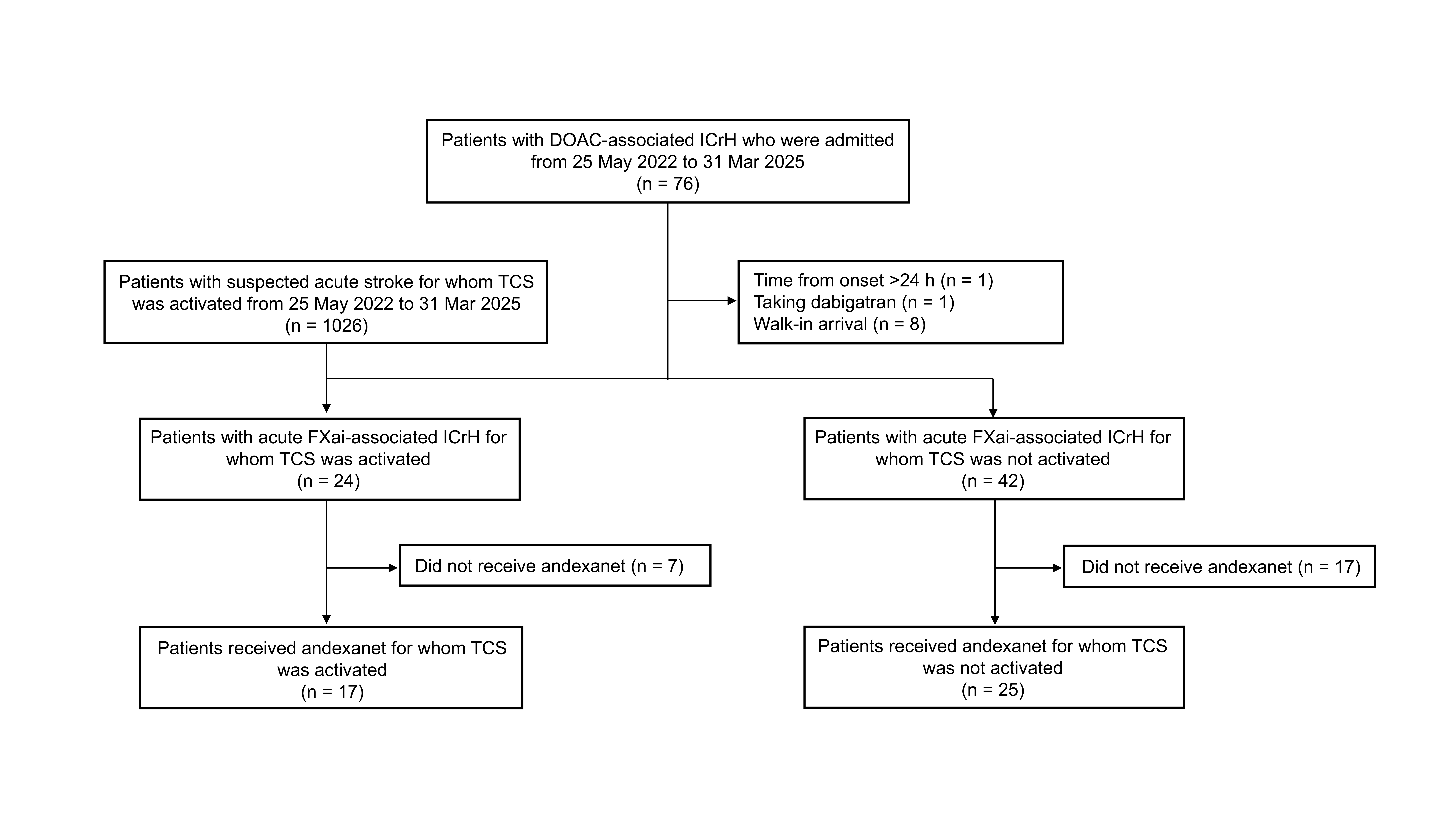

Supplement: Supplementary Figure 2 — Study flowchart. DOAC, direct oral anticoagulant; FXai, Factor Xa inhibitor; ICrH, intracranial hemorrhage; TCS, Task Calculation Stroke. [file Image_2.tif]
