# Supplementary material for: Impact of code stroke on door-to-andexanet administration time for factor Xa inhibitor-associated intracranial hemorrhage: a single-center retrospective study
Source: Front Neurol. 2026 Mar 25;17:1765311. doi: 10.3389/fneur.2026.1765311 (PMC13056841; doi:10.3389/fneur.2026.1765311)
Supplement: Supplementary file 3 [file Data_Sheet_1.pdf]

Supplementary Material

Supplementary Table 1. Patients' characteristics

	TCS-based CS		
Variable	Yes (n = 24)	No (n = 42)	*p*-value
Sex, male	16 (67)	18 (43)	0.08
Age in years	80 (75.5–86)	83.5 (76–89)	0.18
Body mass index	22 (19–26)	21 (18–23)	0.19
Prehospital GCS	13 (8–14)	14 (12–15)	0.15
History of preceding trauma	3 (13)	22 (52)	<0.01
Treatment target disease of FXai			0.72
Atrial fibrillation	17 (71)	33 (79)	
Deep-vein thrombosis	4 (17)	6 (14)	
Other/unclear	3 (13)	3 (7)	
FXai use			0.39
Apixaban	5 (21)	8 (19)	
Rivaroxaban	2 (8)	9 (21)	
Edoxaban	17 (71)	25 (60)	
Arrival time, regular hours	11 (46)	17 (40)	0.67

Data are presented as n (%) or median (interquartile range). Abbreviations: CS, code stroke; FXai, factor Xa inhibitor; GCS, Glasgow Coma Scale; TCS, Task Calculation Stroke.

Supplementary Table 2. The effect of TCS-based CS on door-to-andexanet administration time

Variable	Exp (β)	95% CI	*p*-value
TCS-based CS (yes)	0.54	0.39–0.76	<0.01
Arrival time (regular hours)	0.69	0.52–0.93	0.02
Hematoma volume (per 10-ml increase)	1.03	1.00–1.05	0.03
History of preceding trauma	0.82	0.58–1.15	0.24
Prehospital GCS (per 1-unit increase)	1.03	0.98–1.07	0.29

Exp (β) indicates the rate of decrease in door-to-andexanet administration time. Abbreviations: CI, confidence interval; CS, code stroke; GCS, Glasgow Coma Scale; TCS, Task Calculation Stroke.
